# S05 National Implementation Programmes to promote Active Ageing: Points of Success and Challenges

**DOI:** 10.1093/eurpub/ckae114.217

**Published:** 2024-09-26

**Authors:** Katja Borodulin

**Affiliations:** Age Institute

## Abstract

After retirement, nowadays, we can expect to live from 20 to 40 years of time. For such a long time window, plenty of opportunities should be offered to support active aging. It is surprising how few countries have developed national physical activity programs for older people, particularly knowing the benefits of physical activity for maintenance of functional capacity, prevention of non-communicable diseases and postponing of care and services. Physical activity promotion among older persons has great potential, while it requires more public discussion and sharing the existing good practices.

In this symposium we share three examples of national physical activity programs for older persons. We present their aims, target groups, and main activities. All programs rely on research-based evidence in their implementation, collect results and regularly evaluate their activities. They present action that has increased physical activity, promoted knowledge and education, and improved co-operation. Country examples include issues on scaling-up, sustainability, and communications. Furthermore, challenges and potential solutions in each case are shared and discussed. We discuss and share the take-home-messages and ask the audience to share their views for improved action towards the future.

**Presentations in the symposium include:**

Katja Borodulin (chair and discussant, Age Institute, Helsinki, Finland).

